# Mutagenic Effects of Iron Oxide Nanoparticles on Biological Cells

**DOI:** 10.3390/ijms161023482

**Published:** 2015-09-30

**Authors:** Niluka M. Dissanayake, Kelley M. Current, Sherine O. Obare

**Affiliations:** Department of Chemistry, Western Michigan University, Kalamazoo, MI 49008, USA; E-Mails: nilukamadhumi.m.dissanayake@wmich.edu (N.M.D.); kelley.m.current@wmich.edu (K.M.C.)

**Keywords:** iron oxide nanoparticles, mutagenicity, mammalian cells, bacteria, environmental impact, humic acid, 8-OHdG

## Abstract

In recent years, there has been an increased interest in the design and use of iron oxide materials with nanoscale dimensions for magnetic, catalytic, biomedical, and electronic applications. The increased manufacture and use of iron oxide nanoparticles (IONPs) in consumer products as well as industrial processes is expected to lead to the unintentional release of IONPs into the environment. The impact of IONPs on the environment and on biological species is not well understood but remains a concern due to the increased chemical reactivity of nanoparticles relative to their bulk counterparts. This review article describes the impact of IONPs on cellular genetic components. The mutagenic impact of IONPs may damage an organism’s ability to develop or reproduce. To date, there has been experimental evidence of IONPs having mutagenic interactions on human cell lines including lymphoblastoids, fibroblasts, microvascular endothelial cells, bone marrow cells, lung epithelial cells, alveolar type II like epithelial cells, bronchial fibroblasts, skin epithelial cells, hepatocytes, cerebral endothelial cells, fibrosarcoma cells, breast carcinoma cells, lung carcinoma cells, and cervix carcinoma cells. Other cell lines including the Chinese hamster ovary cells, mouse fibroblast cells, murine fibroblast cells, *Mytilus galloprovincialis* sperm cells, mice lung cells, murine alveolar macrophages, mice hepatic and renal tissue cells, and vero cells have also shown mutagenic effects upon exposure to IONPs. We further show the influence of IONPs on microorganisms in the presence and absence of dissolved organic carbon. The results shed light on the transformations IONPs undergo in the environment and the nature of the potential mutagenic impact on biological cells.

## 1. Introduction

Iron represents the fourth most common element in the earth’s crust and is ubiquitous within nature, industry, and basic consumer products. The prevalence of iron in nature, in its various oxidized forms, in combination with low extraction costs, has made finding potential applications for iron oxide nanoparticles (IONPs) highly attractive. From an industrial perspective, iron oxides are mined to support the production of building materials, pigments, and nutritional supplements. Ninety-eight percent of mined iron oxide is converted to steel for use in consumer products [1]. In addition, to the applications involving steel, IONPs are frequently used as pigments (which are low cost, colorfast, nontoxic and capable of imparting yellow, red, black, or brown color to a wide variety of consumer products); and are used as a food additive, which fortifies foods without altering their color or taste [2,3].

**Figure 1:**
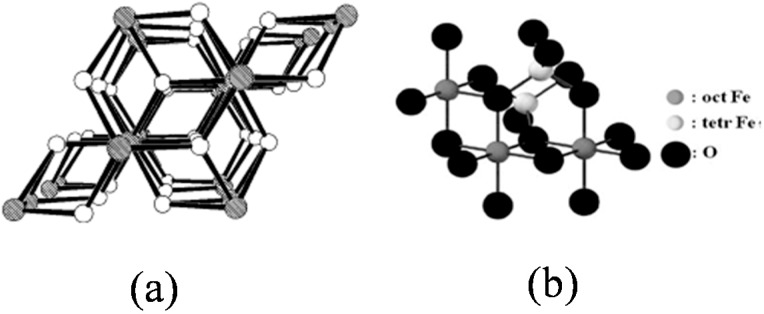

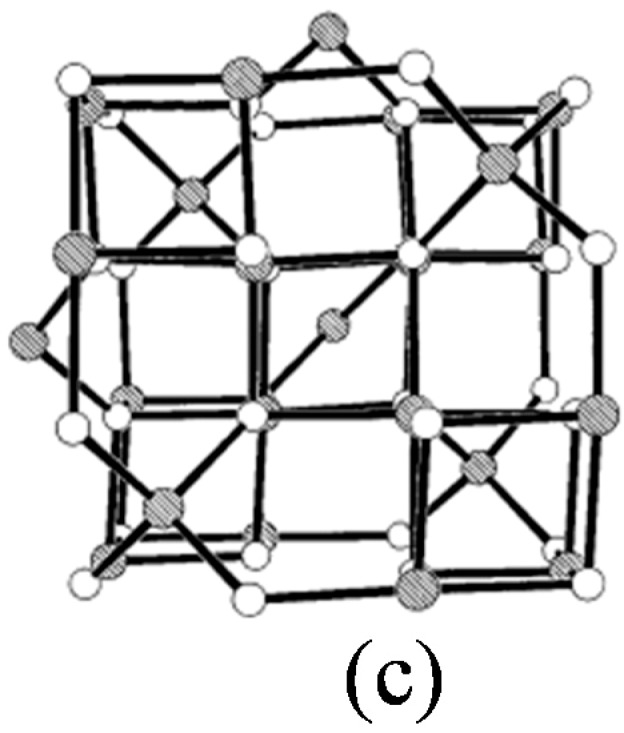
(**a**) Crystal packing of hematite, shaded circles represent Fe and unshaded circles represent O [23]; (**b**) Crystal packing of magnetite, dark large circles represent O, small light circles and small dark circles represent octahedral and tetrahedral coordinated Fe, respectively [24]; and (**c**) Crystal packing of maghemite, shaded circles represent Fe and unshaded circles represent O [23]. Reprinted with permission from reference [24].

New applications for IONPs continue to emerge. Researchers are investigating IONPs as potential drug delivery systems [4], hyperthermia agents [5], magnetic resonance imaging contrast agents [5,6] catalysts for environmental remediation, and much more [7,8,9,10,11,12]. The array of applications associated with IONPs is a result of their tunable properties, which are derived from unique, structurally driven characteristics. There are multiple crystallographic structures exhibited by IONPs that include: magnetite (Fe_3_O_4_), maghemite (γ-Fe_2_O_3_), hematite (α-Fe_2_O_3_), wüstite (FeO), ε-Fe_2_O_3_, and β-Fe_2_O_3_ [13,14,15]. This review will focus on the most common crystallographic structures: magnetite, maghemite, and hematite (Figure 1). Magnetite has an inverse spinel structure and is ferrimagnetic due to the alternating Fe(II) and Fe(III) lattices, which are separated by oxygen atoms that allow for electronic coupling [16,17]. Like magnetite, maghemite is also ferrimagnetic and has an inverse spinel structure [18,19]. Maghemite’s strong ferrimagnetism is derived from lattice vacancies, which give rise to uncompensated electron spins [20]. Hematite, a nanoparticle with a corundum crystal structure, is weakly ferromagnetic due to the coupling between Fe(III) ions across crystallographic planes [21,22].

### 1.1. Potential Applications of Iron Oxide Nanoparticles (IONPs)

In recent years, IONPs have been at the center of promising applications for biomedical research. Iron oxides are widely considered to be non-toxic, and can be collected or manipulated by an applied magnetic field. Early research highlighting their potential for use as MRI counter agents focused on the mechanism by which the body metabolizes IONPs. Studies showed that iron oxides were initially transported to the liver and spleen [25]; however, in less than seven days the excess iron was either excreted or incorporated into the body [25] (in the form of co-factors, hemoglobin, *etc.*).

Studies have shown a variety of biomedical applications that utilize IONPs. These applications include but are not limited to: magnetic resonance imaging, drug delivery, hyperthermia treatment, theranostics, and targeted gene delivery [26,27,28,29]. Like many other bulk materials, the properties of iron oxides change substantially when their particle size is reduced to the nanoscale. Each of the aforementioned applications requires materials that exhibit superparamagnetism, a property exhibited by nanoscale iron oxides [30]. Superparamagnetism allows magnetite and maghemite nanoparticles to exhibit magnetic properties only when subjected to an applied magnetic field, thus making possible superparamagnetic iron oxide nanoparticle (SPION) solutions to be injected and directed toward a target site *in vivo* by the application of an applied magnetic field. Researchers have used SPION solutions to destroy tumors via thermal ablation [31] and have made SPIONs into localizable drug carriers coated with therapeutically relevant compounds [13].

Chemists and material scientists are rapidly developing a wide variety of applications based on the unique properties of IONPs. Such nanoparticles have proven useful in the selective detection of specific gases [32]. For example, hematite thin films have shown promise as selective detectors of gaseous NO_2_ [33]. “Flowerlike” hematite nanoparticles have been used to selectively detect ethanol molecules [34]. Similarly, hematite nanowire sensors possess a high sensitivity and response to carbon monoxide [35]. The selective detection of gases by varied forms of IONPs results from the variation in bandgaps, atom fractions, and exposed crystalline faces inherent in the crystallographic forms [32]. When gases adsorb onto nanoscale sized IONP structures, their resistivity is altered and a proportional change in current is detected [35]. Variation with respect to exposed crystalline faces and atom fractions dictates the level of adsorption of different gases [32].

**Figure 2:**
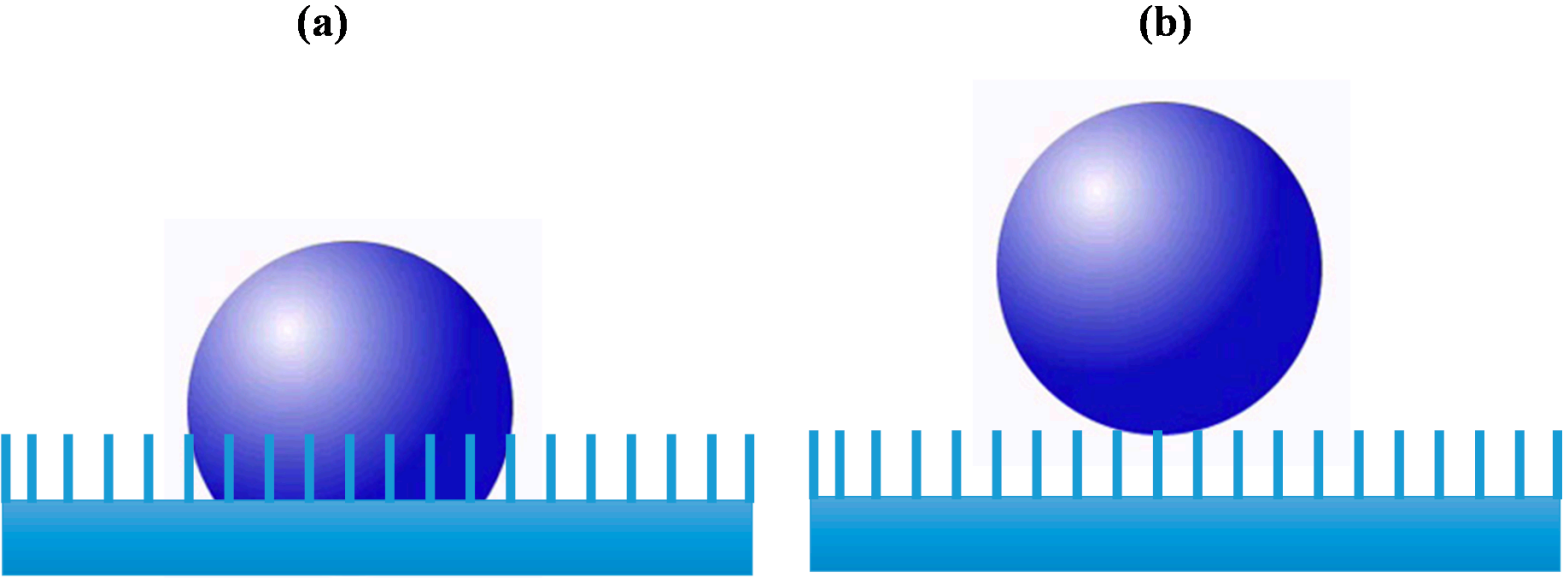
(**a**) Wendzel droplet (occurring when a water droplet merges with a surface) and (**b**) Cassie droplet (occurring when a water droplet is positioned on the surface) above nanosized pockets of ambient air.

Other studies have focused on methods by which synthetic surfaces comprised of precisely configured IONPs, are produced [36]. These synthetic surfaces have finely tuned wetting properties, which are capable of preventing ice build-up [36]. The wetting properties of a surface directly impact its ability to support ice formation. A surface’s wetting properties are controlled, in part, by the surface’s hierarchical roughness at the boundary between the solid and liquid phases [37]. There are two possible equilibrium positions for droplet formation on a rough surface; the Wendzel state, which occurs when the water droplet merges with the surface, as shown in Figure 2a and the Cassie state, which occurs when the water droplet is positioned on the surface above nanosized pockets of ambient air as shown in Figure 2b [37]. The geometric configuration and composition of the surface dictates the most energetically favorable equilibrium position (Wendzel or Cassie) [38]. Researchers have successfully controlled the size and formation of IONP protuberances through the manipulation of an applied magnetic field and by careful selection of IONP stabilizers. IONPs coated with hydrophobic surfactants, which were subjected to stronger magnetic fields during the calcination process produced the most distinct cavities and protuberances [36]. Indirect manipulation of IONP protuberances and cavities has resulted in synthetic ice-phobic surfaces with minimal wettability [36].

The use of IONPs to improve the capacity of lithium ion batteries has been investigated. For example, Wang *et al.* reported the fabrication and testing of an IONP/nitrogen doped aerogel comprised of graphene sheets [39]. The anchored IONPs promote the aerogel’s functionality as an anode by shortening the lithium ion and electron diffusion distance [40]. By crystallographic fusion across graphene sheets, IONPs also promote the formation of a porous structure, which favors electrolyte permeation. These doped aerogels are considered as promising agents for the improvement of battery technologies because they are inexpensive to produce [39,40].

IONPs are also being investigated by a variety of researchers for their utility as agents for environmental remediation. Reports have shown that IONPs (of various forms and bound to various substrates) can be used for the removal of heavy metals from drinking water [41] or for the selective degradation of wastewater dyes [42]. A brief synopsis of these applications is provided below, however, a more detailed description was recently published Xu *et al.* [43].

The removal of heavy metal ions from water supplies is of paramount importance [44]. Heavy metals are not biodegradable, are known to be carcinogenic, and are increasingly being introduced into water supplies and the environment [45]. Multiple methods for the removal of heavy metal ions from water have been developed and include: oxidation/reduction, sedimentation, chemical precipitation, carbon adsorption, membrane filtration and ion exchange [46]. Unfortunately these methods require significant capital both to acquire and maintain. Reports show that specially functionalized SPIONs can be used for the removal of heavy metals from water supplies. Magnetite reduced graphene oxide nanoparticles have proven capable of near complete arsenic removal (99% within 1 ppb) from contaminated drinking water [41]. In another investigation, poly(3,4-ethylenedioxythiophene) coated magnetite nanoparticles were shown to readily conjugate heavy metal ions dispersed within an aqueous solution [46]. In both investigations, the superparamagnetism of IONPs was used to remove heavy metal contaminants (adsorbed on to the SPION surface) by collection of the nanoparticles with an applied magnetic field.

Similarly, researchers are investigating the adsorptive power of IONPs with respect to the collection and disposal of dyes from wastewaters [47]. When introduced into an aqueous environment, wastewater dyes degrade via an oxygen consuming process, which produces chemicals that are suspected to be carcinogenic and disturb the existing ecosystem [48]. To avoid environmental exposure, wastewater dyes must be collected and treated prior to disposal [49]. Mak and Chen successfully collected methylene blue dye by: (1) exposing contaminated solutions to SPIONs; (2) allowing the dye time to adsorb onto the SPION surfaces; and (3) subsequently removing the dye coated SPIONs from solution by application of a magnetic field. After collection, the dye desorbed from SPION surfaces when submerged in ethanol [49]. These researchers have exploited the high surface area to volume ratio and superparamagnetism of SPIONs for wastewater dye binding and collection, respectively [49]. Other researchers have focused on the degradation of wastewater dyes, also using SPIONs. Fenton-like catalytic degradation by these nanoparticles has proven effective in the remediation of wastewater dyes [42]. The nanosized nature of the SPIONs enhances catalytic activity due to significant amounts of exposed surface area, relative to the bulk. Also important is the crystallinity, iron content, and the oxidation state of the SPIONs [48,50]. The crystallinity dictates which faces are most available for catalytic reactions, while the iron content and oxidation state control the type of ions released into solution [50].

### 1.2. IONPs Incomplete Toxicological Profile

Significant research focused on the use of IONPs has stemmed from the ability to generate them inexpensively with controlled size, shape and coatings [51], their magnetic properties, and the considerations that these materials are thought to be non-toxic [39]. However, recent research calls into question the benign nature of IONPs [52,53,54,55]. As growing numbers of consumer products and industrial processes contain nanoparticles, the unintentional release of these substances into the environment is expected, and the impact of these materials is becoming increasingly significant [55,56,57]. The unique properties of nanoscale materials causes concern for their behavior in an environmental setting where they are likely to interact with various chemicals and biological species. Thus, an assessment of IONP toxicity cannot be made based solely upon the toxicological profile of its bulk counterpart [58]; similarly an assessment of environmental toxicity cannot be made in the absence of environmentally significant compounds, such as dissolved organic carbon. Generally speaking, the interaction between cell membranes and nanoparticles is controlled by nanoparticle shape, size, and surface functionalization [59]. There are a multitude of sizes, shapes, and surface functionalized IONPs described in the literature. The wide array of IONPs is one reason why a toxicological profile of these particles has not yet been well documented in the literature; though there are increasing indications that IONPs are not as benign as their bulk counterparts [60].

This review article focuses on IONPs and their impact on the genetic components of biological cells. Increasingly, researchers are finding evidence that IONP exposure can produce mutagenic effects. These interactions directly correlate with DNA alteration and have the potential to damage an organisms development and reproduction. As IONP applications and manufacturing continue to rise, so too will environmental exposure [55]. This review article seeks to highlight research investigating the impact that IONP exposure on the genetic components of various cell lines and bacterial strains.

## 2. Mutagenic Impact of IONPs on Cell Lines

The increase in the manufacturing and use of nanoparticles has led to significant advances in modern technology. Despite the numerous advantages that nanoparticles offer toward modern technology, several concerns exist that render nanoparticles as emerging contaminants. Studies reveal that nanoparticles have adverse effects on biological cells. These adverse effects include mitochondrial damage, oxidative stress, chromosomal and oxidative DNA damage, altered cell cycle regulation and protein denaturation [61,62,63]. However, the mechanisms by which nanoparticles impact toxic effects on biological cells are not well understood. One of the most commonly suggested mechanisms of toxicity is the generation of reactive oxygen species (ROS) by the nanoparticles [64]. More specifically, the interaction of metal oxide nanoparticles, including IONPs with biological cells has led to the observation of different types of DNA damage, including: chromosomal aberrations, DNA strand breakage, oxidative DNA damage and mutations [65].

The preparation of anthropogenic nanoparticles often requires that a stabilizing material is used to coat the surface. The stabilizer protects the nanoparticle from agglomeration, minimizes the rate of surface oxidation, and controls the particle size during the synthesis process. Unstabilized nanoparticles show greater instability, undergo trapping by the immune system, exhibit increased chemical reactivity and undergo oxidation more readily than do stabilized nanoparticles [66]. There are various types of coatings including organic ligands, polymers (natural or synthetic), inorganic molecules, and biological molecules [62]. A study of the cellular uptake of IONPs using different cell lines showed that the nanoparticle uptake efficiency was dependent on the surface coating, irrespective of the cell line used [63]. The study showed that the surface coating could increase the biocompatibility of the nanoparticles and influence IONP toxicity. For example, an *in vitro* study on A3 human T lymphocytes showed that IONPs coated with ligands having terminal carboxylic acid groups exhibited higher cytotoxicity than those coated with ligands bearing terminal amine groups [64]. In another study, citrate coated SPIONs exposed to rat macrophages showed elevated levels of malonyldialdehyde and protein carbonyls that resulted from oxidative stress [67].

Magnetic IONPs including maghemite, hematite, and magnetite have a variety of potential biomedical applications in both diagnostics and therapeutics [65,66]. This is because the iron metabolism is well controlled whereby excess iron is efficiently removed from the body by hepcidin, which is the central regulator of iron homeostasis [68]. However, at the nanoscale, concerns arise as IONPs cause cell damage, including: disruption of cytoskeleton, apoptosis and oxidative stress in both human and mammalian cells [61,62,69,70]. Excess iron exposure has been found to cause elevated ROS generation through the Fenton reaction, resulting in oxidative stress that damages DNA, lipids and proteins, consequently resulting in carcinogenesis [71,72].

When IONPs are used for biomedical applications, control of their interactions with biological systems is a challenge. IONPs immersed within a physiological environment are covered by a layer of proteins forming a “corona” [73]. Blood, which has over 1000 proteins, is often the first physiological environment a nanoparticle interacts with [74]. Protein corona characteristics alter the size, aggregation state, and interfacial properties of IONPs, giving the nanoparticles a biological identity, which differs from their synthetic identity. Upon entering a physiological environment, the surrounding proteins migrate to the particle’s surface by diffusion or by travelling down a potential energy gradient. Protein adsorption on the surface occurs spontaneously if it is thermodynamically favorable. Protein–IONP interactions can be characterized as covalent or non-covalent and may involve rearrangement of interfacial water molecules, or conformational changes in the protein or in the nanoparticle’s surface. The interaction between the protein and the nanoparticle occurs through a special region of the protein known as the “domain” [75]. Essentially, the protein corona is a result of simultaneous adsorption of multiple proteins via protein-nanoparticle or protein-protein interactions. Proteins that adsorb with a high affinity, and are tightly bound form a hard corona. Conversely, proteins that adsorb with a low affinity, and are loosely bound form a soft corona. Hard corona proteins directly interact with the nanoparticle’s surface, while soft corona proteins interact with the hard corona via protein-protein interactions [76,77]. The resultant protein corona, a composite of the hard and soft corona, induces a physiological response when exposed to biomolecules, biological barriers, and cells [77]. It has been recognized that protein corona formation is ubiquitous and independent of NP nature [78]. Researchers have uncovered protein corona formation in several studies. Rapid protein corona formation was observed in the presence of silica and polystyrene nanoparticles [79], and long lived corona were found on nanoparticles exposed to serum or plasma [80]. Other studies have investigated the biocompatibility of nanoparticles upon corona formation [81] as well as the nanoparticles aggregation and cell viability within the culture medium [82]. Consequently, reports have shown that the protein corona can alter the interactions between nanoparticles and produce aggregation, leading to the need and use of polymeric surface stabilizer that protect the nanoparticle surface from protein corona formation [57,83,84,85,86]. Furthermore, stable polymer coated magnetic nanoparticles were reportedly used in biomedical applications [6,12] and for wastewater remediation [46].

Nanoparticle coating characteristics and size are known to impact the cellular uptake of IONPs [84]. It is important to recognize that though the doses of IONPs with which cells were treated are presented, these values do not necessarily represent the amount of internalized iron [84]. This is somewhat problematic, as there is good reason to suspect that the amount of internalized IONPs (governed not only by NP dose, but also by coating composition, coating thickness, and media composition [85]) impact reported levels of mutagenicity. Thus, the dose of nanoparticles impacts, but may not be equivalent to, cellular internalization of IONPs [86]. Additionally, levels of IONP internalization mediate the observed levels of toxicity and mutagenicity. The toxicity and mutagenicity associated with various IONPs presented in this review are categorized by IONP dose because this is the current standard of reporting within the literature. However, readers are cautioned to also consider the role that cellular uptake of IONPs may have played. Researchers interested in adding to the experimental literature are encouraged to consider indicating the dose of IONPs applied and determining the level of cellular uptake using the method suggested by Galimard *et al.* [86].

### 2.1. General ROS Generation and DNA Damage Mechanisms

The cellular oxidation mechanism involves a sequence of electron and proton transfer reactions in which, molecular oxygen is reduced to water and ATP is synthesized. However, in most cases a small amount of the molecular oxygen does not undergo complete reduction to water but instead may be converted into superoxide anion radicals (O_2_·^−^) or other oxygen-based ROS including hydroxyl radicals (·OH), singlet oxygen (^1^O_2_) and hydrogen peroxide (H_2_O_2_). These ROS play an important role in cellular signaling systems. Except for this cellular oxidative stress, copper and iron participate in single electron oxidation-reduction reactions leading to ROS formation [87]. ROS generation induced by nanoparticles has a great impact on mutagenicity since DNA is a critical cellular target of ROS [72].

Nanoscale sized metal and metal oxide particles with redox characteristics can enhance the formation of ROS by acting as catalysts in ROS production reactions. For iron, two types of reactions known as the Fenton reaction and the Haber–Weiss reaction are shown in Equations (1)–(3). Iron ions released in to the cytosol as a result of lysosomal enzymatic degradation participate in these reactions producing radicals [72], especially when nanomaterials are in a suspending medium or a biological system. This leads to the generation of ionic species promoting toxicity [88].

Fenton reaction:

Fe^2+^ + H_2_O_2_ → Fe^3+^ + ·OH + :OH^−^ (1)

Haber–Weiss cycle reaction:

Fe^3+^ + ·O_2_^−^ → Fe^2+^ + O_2_ (2)

Fe^2+^ + H_2_O_2_ → Fe^3+^ +·OH + OH^−^ (3)

**Figure 3:**
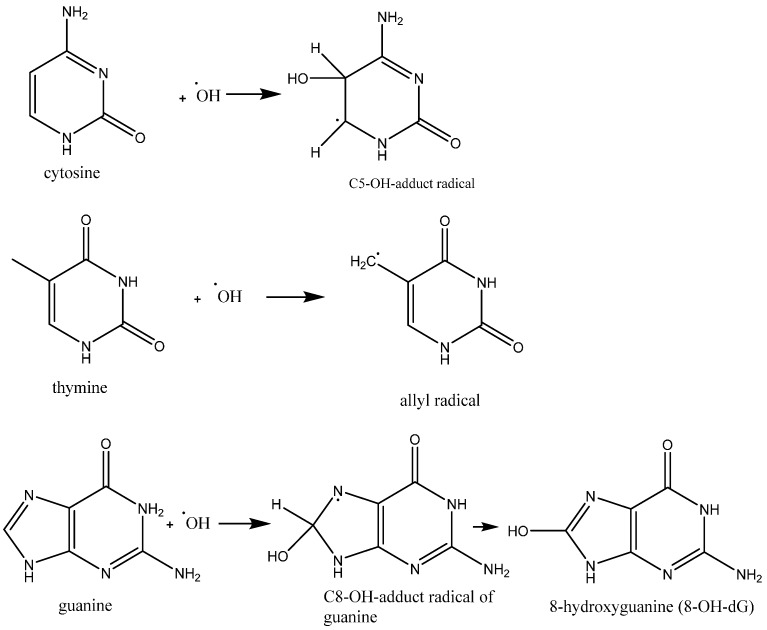
Reactions of ·OH radical with DNA nitrogenous bases cytosine, thymine and guanine.

**Figure 4:**
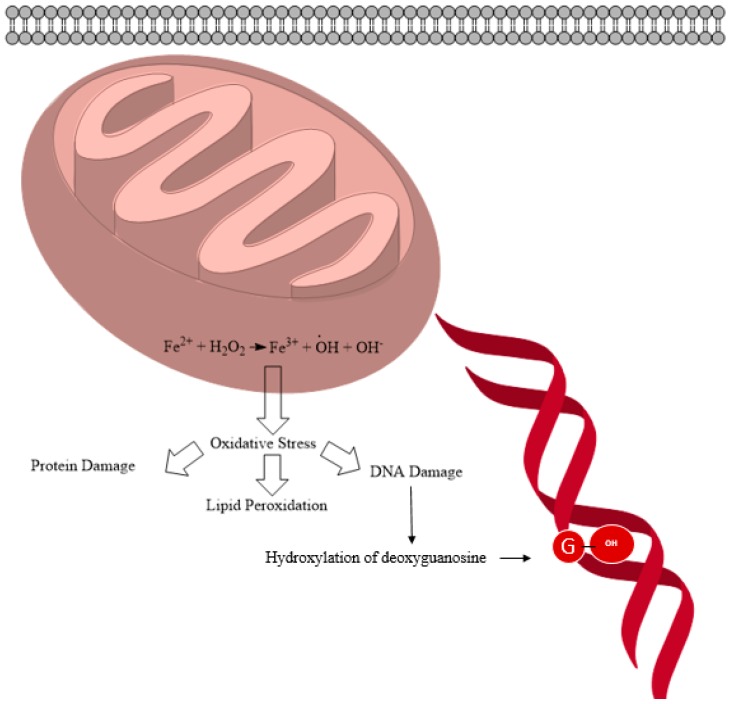
Schematic diagram showing the fate of iron oxide nanoparticles (IONPs) inside a cell leading to mutagenicity, hydroxylation of the deoxyguanosine (pictured as a circled “G”).

**Figure 5:**
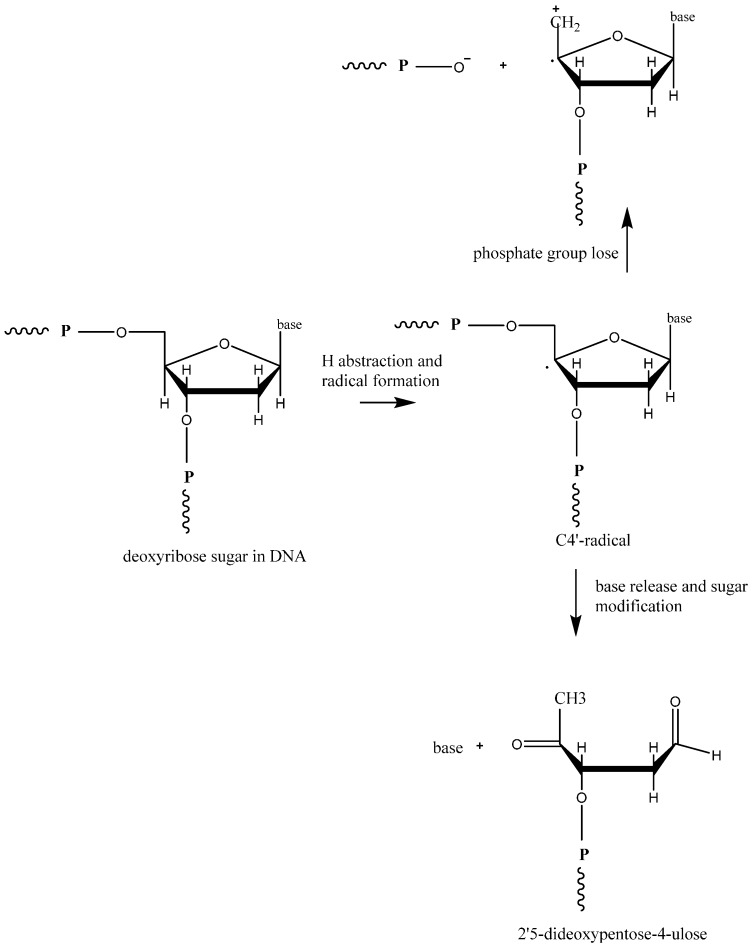
Sugar-phosphate backbone damaging reactions on DNA, including C4ʹ-radical formation from deoxyribose sugar, base release, sugar modification, and loss of phosphate group.

Studies estimate that a human cell is exposed to approximately 1 × 10^5^ oxidative hits per day from hydroxyl radicals and other ROS [89,90]. The hydroxyl radical can react with purine and pyrimidine bases as well as with the deoxyribose-phosphate back bone, damaging the molecule [89,90]. More than 100 products have been identified resulting from oxidative damage. ROS-induced DNA damage accounts for DNA single and double strand breakage as well as purine, pyrimidine or deoxyribose modifications, and DNA cross-link formation [91,92]. Hydroxyl radicals have been shown to add to the double bond of the pyrimidines and purines at diffusion-controlled rates. The second order rate constant for these types of reactions ranges from 4.5 × 10^9^–9 × 10^9^ M^−1^·s^−1^ [93]. These reactions produce the hydroxyl-adduct radical or the allyl radical product of the relevant base (Figure 3). These radical products are further oxidized or reduced depending on their redox environment, redox properties and reactants to yield a variety of products [94,95]. When a hydroxyl radical attacks guanosine, 8-hydroxyguanosine (8-OHdG) is produced as depicted in Figure 4. In nuclear and mitochondrial DNA, 8-OHdG acts as a free radical and further induces oxidative damage within its region as shown in Figure 5. Therefore, 8-OHdG acts as a biomarker of oxidative stress and DNA damage. It also acts as a risk factor for many diseases, including cancer [96,97]. This modification occurs once for every 10^5^ human guanine residues [98].

Hydroxyl radicals can abstract a H atom from the sugar component of DNA as shown in Figure 5 at rate constant of 2 × 10^9^ M^−1^·s^−1^. The C4ʹ-radical of the deoxyribose sugar in DNA can undergo several reactions, which lead to DNA strand breakage. The breakage of the strand may be due to release of free modified sugars or sugars with terminal groups of broken DNA strands. 2,3-dideoxypentos-4-ulose and 2,5-dideoxypentose-4-ulose act as free modified sugars. In the absence of oxygen, the C4ʹ-radical of the deoxyribose sugar can lose a phosphate group on either side of the DNA leading to strand breakage [87,93]. Apart from these modifications, elimination of modified bases due to weakened glyosidic bonds occur often and are referred to as base free sites (apurinic sites/aprydimidinic (AP) sites) [93]. Upon exposure of DNA to free radicals, like H_2_O_2_ or ionizing radiation, DNA-protein crosslink formation occurs. In mammalian cells, chromatin thymine-tyrosine crosslinking has been observed [99,100].

In recent years, there have been several studies reporting the mutagenic effects of IONPs. Table 1 summarizes the investigations focusing on IONPs and their impact on DNA. Most studies compare the impact of bare IONPs and coated IONPs on various cell lines. Different mammalian cellular models have been studied, these include: blood cells, vascular cells, stromal cells, reproductive cells, lung cells, liver cells, skin cells, brain cells, and cancer cells. In many of these studies, DNA damage is observed. Though researchers have begun to investigate the impact of IONPs on mammalian cellular genomes, little attention has been paid to the impact of IONPs on bacterial genomes.

Common methods to detect DNA damage include: the comet assay [101], micronucleus (MN) test [102], and 8-OH-dG detection. Furthermore, DNA damage can be accessed via the enzymatic digestion of DNA [103]; where products are identified by high performance liquid chromatography (HPLC) or (electrochemical HPLC) EC-HPLC. Acidic hydrolysis is another method by which DNA damage may be detected, where free bases are liberated and isolated using HPLC and subsequently identified with gas chromatography mass spectrometry (GC–MS) [72].

**Table 1:** IONP and DNA damage evidences from past studies.

IONP Type and Surface Modification	Characterization and Size	Cellular Model/Organism	Impact on Cells	Ref.
Silica-coated, dithiocarbamate functionalized Fe_3_O_4_ NP alone and NP co-exposure with Hg	Particle sizes 100 nm (DLS)	European eel (*Anguilla anguilla* L.) erythrocytes	IONP-Hg complex can eliminate DNA damage which is induced by IONP or Hg alone. IONP alone is more capable of inducing mutagenicity than Hg.	[104]
Nanoscale and bulk materials of Fe_3_O_4_	Nanoscale = 29.75 nm, bulk 2.15 µm (TEM)	Rat leucocytes and bone marrow cells	Showed no evidence of DNA damage by comet assay or micronucleus (MN) test for any of the tested particles.	[105]
Uncoated Fe_3_O_4_ (magnetite), and uncoated γ-Fe_2_O_3_ (maghemite) Dextran coated ultra-fine superparamagnetic Fe_3_O_4_ (dUSPION), dextran coated ultra-fine superparamagnetic γ-Fe_2_O_3_ (d USPION2)	Particle size = 1 nm (TEM)	Human lymphoblastoid cell line (MCL-5)	γ-Fe_2_O_3_ dUSPION2 showed significant DNA damage at a concentration of 4 µg·mL^−1^ and higher, having accumulated oxidative base lesions (including 8-OH-Gua).	[106]
Uncoated and oleic acid coated Fe_3_O_4_ (magnetite) NPs	Particle size = 9 nm	Human lymphoblastoid TK6 cells	No genotoxic effect was observed for the bare particles. However, an increased base oxidation was observed for oleic acid coated particles after 2 and 24 h of treatment.	[107]
Fe_2_O_3_ NPs	X-ray diffraction particle crystal diameter 31.1 nm	Human lymphoblastoid cells (TK6), Chinese hamster ovary cells (H9T3)	Iron oxide samples of 10 and 20 g/mL produced DNA tail percentages 17% and 20%, respectively, after 4 h in TK6 cells. The same two concentrations of iron oxide samples produced DNA tail percentages 33% and 48%, respectively, after 24 h in H9T3 cells.	[108]
Unfunctionalized Au@Fe_3_O_4_ Janus particles and functionalized particles with NH_2_	Particle core sizes, gold domain 3.5 nm and iron oxide domain 16 nm (TEM)	Human microvascular endothelial cells	DNA damage was observed for unfunctionalized Janus particles, compared to NH_2_ functionalized particles.	[109]
Fe_3_O_4_ (magnetite) and Fe_3_O_4_–poly(l-lactide)–poly(ethyleneglycol)–poly(l-lactide) magnetic microspheres (Fe_3_O_4_–PLLA–PEG–PLLA MMPs)	30 nm	Mouse fibroblast cell line (L929) Chinese hamster ovarian cell line (CHO-K1)	Fe_3_O_4_–PLLA-PEG-PLLAMMPS caused less DNA damage than Fe_3_O_4_ particles.	[110]
Fe_3_O_4_ (magnetite) NPs, tetraethyl orthosillicate (TEOS) coated IONP, 3-aminopropyl trimethoxy silane (APTMS) coated IONP and TEOS/APTMS coated IONP	HR-TEM bare particle size 10 ± 3 nm. TEOS coated particles 100–150 nm, APTMS coated 10 ± 4 nm, TEOS/APTMS coated 100–150 nm	Human normal fibroblast and fibrosarcoma cells	Both cell types showed a dose dependent increase in DNA tail size. Bare and TEOS coated NPs showed no extensive or dose dependent DNA damage (lower than 5% damage at 1000 g/L). APTMS and APTMS/TEOS coated NPs produced a significant dose dependent toxicity when exposed to normal cells.	[26]
Maghemite (γ-Fe_2_O_3_) γ-Fe_2_O_3_ coated with poly-l-lysine, d-mannose, poly(*NN*-dimethylacrylamide)	Number-average particle diameter γ-Fe_2_O_3_ 6 nm, PLL-γ-Fe_2_O_3_ 5.5 nm, mannose γ -Fe_2_O_3_ 7 nm, PDMAAm-γ-Fe_2_O_3_ 7.5 nm (TEM)	Human bone marrow mesenchymal stromal cells from two donors (hBMSCs-1–12 years and hBMSCs-2–54 years)	hBMSCs-2 showed more toxic effects upon exposure to IONPs than did hBMSCs-1. In hBMSCs-2, only PDMAAm-γ-Fe_2_O_3_ and γ-Fe_2_O_3_ particles increased DNA damage after 72 h of exposure to NPs.	[111]
Bare superparamagnetic magnetite (SPION) and poly(vinyl alcohol) PVA coated SPION	Particle size 4.5 nm (TEM)	Mouse fibroblast adhesive cells (L929)	Cells exposed to bare particles showed evidence of cytotoxicity after 24 h. No toxic effect for the coated particles was observed, even after 72 h of exposure. DNA damage is believed to be the reason behind apoptosis.	[112]
Bare SPION (magnetite) citrate coated, tetraethyl orthosilicate (TEOS) coated, 3-aminopropyl trimethoxy silane (APTMS) coated and TEOS/APTMS coated IONP (T-A)	Bare particle size 10 nm. Citrate coated particle 10 nm, TEOS coated particle S 100–150 nm, APTMS coated 10 nm, TEOS-APTMS coated 100–150 nm	Murine fibroblast cell line (L-929 from mouse subcutaneous connective tissue)	No extensive or dose dependent DNA damage was observed for the cells treated with bare and TEOS treated particles. SPIONS modified with APTMS and T-A showed a dose dependent mutagenicity. Cells treated with 200 ppm citrate modified SPIONS showed significant DNA damage.	[113]
Zero valent iron NPs (nZVI) with Na acrylic co-polymer	Particle size 50 nm (TEM)	*Mytilus galloprovincialis* sperm	DNA strand breakage was observed after exposure for 2 h.	[114]
Fe@Fe_2_O_3_ core-shell nanonecklace with MWCNT	Diameter of nanonecklace 50–150 nm (SEM)	Herring sperm DNA	DNA damage was observed by monitoring the DPV (Differential Pulse Voltammetry) response of an electrochemical indicator Co(phen)^3^ or Ru(NH_3_)_6_^3+^.	[115]
Fe@Fe_2_O_3_ core-shell nanonecklace and Au NPs	High magnification SEM image revealed diameter 50–150 nm	Hering sperm DNA	DNA damage was detected within 5–10 min of incubation with cathodic treatment.	[116]
Fe_3_O_4_ (magnetite) microparticles and nanoparticles, Fe_2_O_3_ microparticles and nanoparticles	Particle sizes for Fe_3_O_4_ nano-sized 27 ± 8 nm and micro-sized 156 ± 82 nm, Fe_2_O_3_ nano-sized 35 ± 14 nm and micro-sized 147 ± 48 nm (TEM)	Syrian Hamster embryo cells	No significant DNA damage or micronucleus formation was observed in any cell samples exposed to the iron oxide NPs.	[117]
Fe_3_O_4_ (magnetite) NPs	Particle size 12.5 ± 4.45 nm (TEM)	Mice lung imprinting control region	Significant DNA damage in magnetite treated mice lung cells was observed. Mice treated with low dose magnetite showed a two-fold mutant frequency relative to the control. High dose treated mice showed a three-fold mutant frequency relative to the control.	[60]
Fe_3_O_4_ (magnetite) NPs	Average particle size 10 nm (TEM)	A549 Human lung epithelial cells	8-OH-dG levels increased by 8- and 14-fold above the control with 10 and 100 g/L NPs, respectively.	[28]
Hematite (α-Fe_2_O_3_)in three sizes, Hem-nano, Hem-submicro Hem-micro	Rhombohedral hematite α-Fe_2_O_3_ (XRD) Particle sizes: nano (93 nm), sub-micro (260 nm), micro (1600 nm) from TEM	Human lung epithelial cells (A549), murine alveolar macrophages (MH-S)	No DNA damage was induced by hematite NPs.	[118]
Fe_2_O_3_ microparticles and nanoparticles, Fe_3_O_4_ (magnetite) microparticles and nanoparticles	Particle sizes: Fe_2_O_3_ micro (0.15–1 µm) and nano (30–60 nm) sizes, Fe_3_O_4_ micro (0.1–0.5 µm) and nano (20–40 nm) (TEM)	Human alveolar type II like epithelial cells (A549)	By the comet assay Fe_2_O_3_ and Fe_3_O_4_ caused small but significant increases in DNA damage. In terms of oxidative damage, only Fe_3_O_4_ produced significant DNA damage. The authors report that nanoparticles were higher in oxidative capacity than their micrometer sized particle counterparts.	[119]
Fe_2_O_3_ (hematite) NP	No information provided	human lung cells: IMR 90 (human bronchial fibroblasts) and BEAS-2B cells	After 24 h exposure to Fe_2_O_3_ NPs, IMR-90 cells showed DNA-breakage at concentrations of 10 and 50 μg/cm^2^; then in BEAS-2B cells DNA breakage was observed at 50 μg/cm^2^ Fe_2_O_3_ NPs.	[120]
Fe_3_O_4_ (magnetite) NPs	X-ray diffraction-crystalline 25.27 nm particles, TEM showed polygonal shaped particles with a diameter of 24.83 nm	Human skin epithelial (A431) and lung epithelial (A549) cells	A positive correlation in DNA damage and ROS generation in A431 and A549 cells was observed.	[27]
CuZnFe_2_O_4_, Fe_3_O_4_ (magnetite), Fe_2_O_3_ NPs	No information provided	Type II epithelial cells (A549)	By the comet assay: no DNA damage was observed for IONPs (Fe_3_O_4_, Fe_2_O_3_), but DNA damage was observed for CuZnFe_2_O_4_. In terms of oxidative damage, Fe_3_O_4_ produced oxidative DNA lesions as did CuZnFe_2_O_4_.	[63]
Fe_3_O_4_ (magnetite) NPs	Particle size = 8 nm (TEM)	Human hepatocyte (HL-7702 cell line)	Cells showed nuclear condensation and chromosomal DNA fragmentation after NPs exposure.	[121]
Fe_2_O_3_ NPs	Particle size = 50 nm	Human hepatoma Hep G2 cells	Concentration and time dependent DNA damage was observed.	[53]
Fe_3_O_4_ (magnetite) NPs	Particle size = 35 nm (TEM)	Mice hepatic and renal tissue	In liver tissue there was significant increase of 8-OH-dG levels for the highest dose of NPs (40 mg/kg). In kidney tissues damage was shown for a dose of 20 mg/kg NPs. A significant DPC (DNA-protein crosslinks) coefficient was observed for: (a) a dose of 40 mg/kg NPs in liver tissue and (b) a dose of 10 mg/kg NPs in kidney tissue.	[122]
Ultra small superparamagnetic magnetite IONPs (USPIO NPs, Fe_3_O_4_) oleic acid coated USPIO NPs	Particle sizes 8 ± 3 nm (TEM) 14–15 nm (DLS)	Human cerebral endothelial cells (HCECS)	Single and double DNA strand breaks and alkaline labile sites were detected.	[123]
Aminosilane-coated IONPs (AmS-IONPs) and COOH-AmS-IONPs	No information provided	Mouse brain microvessel endothelial cell line and mouse astrocytes and neurons	No toxicity was observed for any of the particles in brain endothelial cells. At high concentrations neurons displayed a toxicity to AmS-IONPs and astrocytes displayed a toxicity to COOH-AmS-IONPs.	[124]
Fe_2_O_3_ NPs	Particle size ranged between 19.56–48 nm (TEM)	Human breast cancer cell line (MCF-7)	Gradual nonlinear DNA damage was observed as NP dose and exposure time were increased. 60 µg/mL IONP conditions produced the most DNA damage.	[52]
Epidermal growth factor receptor (EGFR) targeted hybrid plasmonic core-shell iron oxide (maghemite) gold NPs (225 NP)	TEM and DLS particle size 73 ± 35 nm	Human HCC827 lung cancer cell line	An increase in DNA strand breaks for 225 NP treated cells was observed (relative to all other treatment conditions). DNA strand breaks were assessed by tracking the levels of phosphorylated H2AX expression. A slight increase in phosphorylated H2AX was also observed in cells treated with: (1) AuFe; (2) 225-Ab (antibody) alone; (3) a mixture of NP and 225-Ab relative to untreated cells.	[125,126]
Bare Fe_3_O_4_(magnetite)/SiO_2_ NPs, Fe_3_O_4_ amine-silane surface modified, Fe_3_O_4_ sulfonate-silane surface modified	TEM, DLS Fe_3_O_4_ core 12 ± 2 nm, SiO_2_ shell thickness 7 ± 1.5 nm, total diameter 26 ± 2.9 nm	Human cervix carcinoma cells (HeLa cells), Human lung carcinoma cells (A549)	Bare NPs increased DNA damage in terms of tail length and DNA percentage in tail relative to passivated NPs, which showed results similar to the control.	[127]
Fe_3_O_4_ (magnetite) NPs	No information provided	HeLa cells	After exposure of 50 µg/mL NPs, no significant change was observed in tail length. 100–200 µg/mL concentrations however, showed increased tail length and DNA percentage in the tail.	[128]
Fe_3_O_4_ (magnetite) and Fe_2_O_3_ NPs	Particle size < 50 nm (TEM)	Vero cell line (C1008), bacterial strains *Salmonella typhimurium* (TA98), *S. typhimurium* TA 100, TA 1535, TA 1537, and *E. coli* WP2uvrA	No change is observed in the number of revertant colonies in IONP treated groups or the negative control. The positive control showed mutagenicity. IONPs do not induce mutagenicity in strains *S. typhimurium* TA 100, TA 1535, TA 1537, and *E. coli* WP2uvrA.	[129]

Ref., References.

### 2.2. Vascular System and Blood Cells

The effect of IONPs on blood, human, rat and eel blood cells were carried out in an effort to determine the impact of these IONPs. European eel (*A. Anguilla* L.) erythrocytes were exposed to silica-coated, dithiocarbamate functionalized Fe_3_O_4_ (magnetite) with Hg co-exposure to determine the interference of Hg on functionalized NPs [104]. Erythrocytic nuclear abnormalities (ENA) were evaluated at times (2, 4, 8, 16, 24, 48 and 72 h) progressively. ENA increase was observed only for IONP under early (2, 4 and 8 h) and late (16, 24, 48 and 72 h) exposure times. IONPs in the presence of Hg showed no ENA increase, proving that the IONP-Hg complexation reduced or eliminated DNA damage. When exposed to IONPs or Hg alone a progressive increase in 8-OHdG levels was observed over time [104]. Rat leucocyte cells and bone marrow cells were exposed to bulk Fe_3_O_4_ and Fe_3_O_4_ (magnetite) nanoparticles [105]. Rats were treated with 500, 1000 and 2000 mg/kg Fe_3_O_4_ bulk and Fe_3_O_4_ nanoparticles and sampled at 6, 24, 48 and 72 h to be assessed by the comet assay. The results showed no significant DNA damage in the comet assay or the MN test.

A human lymphoblastoid cell line, MCL-5, was exposed to the following nanoparticles: uncoated Fe_3_O_4_ (magnetite), and uncoated γ-Fe_2_O_3_ (maghemite), dextran coated ultra-fine superparamagnetic Fe_3_O_4_ (dUSPION), dextran coated ultra-fine superparamagnetic γ-Fe_2_O_3_ (dUSPION2), [106]. The cells were exposed to a range of concentrations (1–100 µg/mL) of Fe_3_O_4_ (dUSPION), γ-Fe_2_O_3_ (dUSPION2), uncoated Fe_3_O_4_, and uncoated γ-Fe_2_O_3_ in a 1% serum medium for 24 h. γ-Fe_2_O_3_ (dUSPION2) NPs induced significant micronuclei formation at a concentration of 4 µg/mL and higher. Dextran coated Fe_3_O_4_ (dUSPION), uncoated Fe_3_O_4_, and uncoated γ-Fe_2_O_3_ induced no micronuclei formation and no DNA damage. The same human lymphoblastoid cell line, TK6, was tested with Fe_2_O_3_ nanoparticles (concentrations of 5, 10 and 20 µg/mL) in a separate study [108]. DNA damage was detected with a high throughput assay using a comet chip. IONP concentrations of 10 and 20 g/mL resulted in 17% and 20% DNA damage, respectively, after 4 h of exposure. The same cell line TK6, was exposed to Fe_3_O_4_ and oleic acid coated Fe_3_O_4_. No genotoxic effects were observed for the bare particles, however an increase in base pair oxidation was observed in the case of oleic acid coated nanoparticles after 2 and 24 h of treatment [107].

Landgraf *et al.* exposed human microvascular endothelial cells to unfunctionalized Au@Fe_3_O_4_ Janus particles and amine-functionalized Janus particles [109]. The cells were exposed to different Janus particle concentrations 1, 10, or 50 µg/mL for 24 h at 37 °C. DNA damage, as assessed by the comet assay, was observed in cells treated with unfunctionalized Janus particles, but was not observed in amine-functionalized Janus particles.

### 2.3. Fibroblast and Stromal Cells

Human and mouse fibroblast/stromal cells have been incubated with several kinds of IONPs including magnetite, maghemite, hematite Fe_3_O_4_, Fe_2_O_3_, and SPION to reveal their effects. In a study with human normal fibroblast cells and Fe_3_O_4_ (magnetite) NPs, cells were exposed to concentrations 100, 200, 1000 µg/mL of bare magneitite nanoparticles, tetraethyl orthosillicate (TEOS) coated IONPs, 3-aminopropyl trimethoxy silane (APTMS) coated IONPs and TEOS/APTMS coated IONPs [26]. Bare and TEOS coated nanoparticles showed no extensive or dose dependent damage to DNA as tested using the comet assay. However, nanoparticles modified with APTMS and TEOS/APTMS showed significant dose dependent DNA damage. Another study exposed maghemite (γ-Fe_2_O_3_) with different coatings to human bone marrow mesenchymal stromal cells. Bare maghemite (γ-Fe_2_O_3_), maghemite (γ-Fe_2_O_3_) coated with poly-l-lysine, d-mannose, poly(*NN*-dimethylacrylamide) were exposed to cells from two donors (hBMSCs-1–12 years old and hBMSCs-2–54 years old) [111]. The cells were incubated for 72 h in a nanoparticle suspension of 15.4 µg/mL in a culture medium. The washed nanoparticles were incubated in a fresh medium for 72 h. Sample hBMSCs-2 exhibited a higher sensitivity and level of toxic effect than did sample hBMSCs-1. In hBMSCs-2, only PDMAAm-γ-Fe_2_O_3_ and γ-Fe_2_O_3_ particles induced an increase in DNA damage after 72 h of exposure. Human bronchial fibroblasts (IMR 90) and Fe_2_O_3_ nanoparticles were used to examine the toxic effects [120]. The cells were exposed to nanoparticles for 24 h. DNA-breakage was observed at concentrations of 10 and 50 μg/cm^2^.

Several studies have used mouse/murine stromal cells and different types of IONPs. Bare SPIONs and poly(vinyl alcohol) PVA coated SPIONs and mouse fibroblast adhesive cells (L929) were used in this study [26]. The cells were exposed to SPIONs for 24, 48, 72, h and 100 µL of 3-(4,5-dimethylthiazol-2-yl)-2,5-diphenyltetrazolium bromide (MTT) were added to wells and incubated. Cells exposed to bare nanoparticles showed evidence of cytotoxicity after 24 h. No toxic effect was observed for the coated particles, even after 72 h of exposure. DNA damage is believed to be the reason behind the observed apoptosis. Murine fibroblast cell line (L-929 from mouse subcutaneous connective tissue) and SPION particles have been used in a different study [113]. The cells were exposed to bare SPION, citrate coated SPION, tetraethyl orthosilicate (TEOS) coated SPION, 3-aminopropyl trimethoxy silane (APTMS) coated SPION and TEOS/APTMS coated SPION (T-A). The cells were exposed to increasing SPION concentrations (200–1000 ppm) for 24 h. Fresh cultures and 10% DMSO treated cell cultures were used as negative and positive controls, respectively. No extensive or dose dependent DNA damage was observed for the cells treated with bare and TEOS-coated nanoparticles. Interestingly, SPIONs modified with APTMS and T-A showed a dose dependent mutagenecity. Cells treated with 200 ppm citrate modified SPIONs also showed significant DNA damage.

### 2.4. Reproductive Cells

There are a few studies that have investigated the effects of IONPs on reproductive cells. In these studies ovarian cells, sperm cells and embryo cells were exposed to IONPs. Two studies were carried out using Chinese hamster ovarian cells with IONPs. In one of the studies cell line H9T3 was exposed to Fe_2_O_3_ nanoparticles at a variety of concentrations (5, 10, 20 µg/mL). Concentrations 10 and 20 g/mL resulted in 33% and 48% DNA damage, respectively, after 24 h in H9T3 cells [108]. In the other study, Fe_3_O_4_ (magnetite) and Fe_3_O_4_–poly(l-lactide)–poly(ethylene glycol)–poly(l-lactide) magnetic microspheres (Fe_3_O_4_–PLLA–PEG–PLLA MMPs) were exposed to CHO-K1 cells [110]. Ovarian cells, (1 × 10^6^) were exposed to different concentrations of MMP suspensions for 24 h. Untreated cells functioned as the negative control, while cells treated with 200 ng/mL of Mytomycine (MMC) functioned as the positive control. Ultimately, Fe_3_O_4_-PLLA-PEG-PLLA MMPS exposed cells exhibited less DNA damage than did cells exposed to bare Fe_3_O_4_ nanoparticle.

Two studies have used *Mytilus galloprovincialis* sperm cells and Hering sperm cells to observe the fate of sperm DNA upon exposure to two different types of IONPs. Zerovalent iron nanoparticles (nZVI) with Na acrylic co-polymer were exposed to *Mytilus galloprovincialis* sperm cells [114]. Sonicated fresh stock solutions of nanoparticles were added to sperm cell suspensions yielding 0.1, 1, 10 mg·L^−1^ concentrations. After the sperm cells were exposed to the nanoparticles for 2 h DNA strand breakage was revealed by the comet assay [114]. Hering sperm DNA were exposed to two types of nanoparticles: (a) Fe@Fe_2_O_3_ core-shell nanonecklace with MWNT [115] and; (b) Fe@Fe_2_O_3_ core-shell nanonecklace and AuNPs [116]. In both studies, DNA damage was detected using differential pulse voltammetry (DPV). This method was capable of detecting DNA damage within 5–10 min of incubation [115]. Syrian Hamster embryo cells were exposed to nano and micro Fe_3_O_4_ (magnetite), as well as nano and micro Fe_2_O_3_ particles in a different study [117]. Cell cultures in 21 cm^2^ dishes were treated with the following particle concentrations for 24 h: 10, 25, 50 µg/cm^2^. Then, those were used in the comet assay and MN test. No significant DNA damage or micronucleus formation, in any cell samples exposed to the IONPS was observed.

### 2.5. Lung Cells

There are several studies that have been carried out using lung cells and IONPs. In most of these cases human and mouse cell models have been studied. Mice lung imprinting control region cells and magnetite nanoparticles were used in a particular study in which the mice were treated with 0.05–0.2 mg/animal of IONPs [60]. The mice treated with a low dose of magnetite showed a two-fold mutant frequency increase relative to the control, while the high dose treated mice showed a three-fold increase relative to the control.

Human lung cell types tested for IONPs toxicity include lung cell BEAS-2B type and lung epithelial cells (A549). BEAS-2B cells were exposed to Fe_2_O_3_ nanoparticles for 24 h. DNA-breakage was observed at concentrations of 50 μg/cm^2^ in BEAS-2B cells [63,119]. Reports have shown tests of the toxic effects of IONPs on lung epithelial cell line A549 [28,60,118]. Totsuka *et al.* exposed magnetite nanoparticles to A549 human lung epithelial cells. The cells were incubated with the following magnetite nanoparticle concentrations for 72 h at 37 °C: 0, 1, 10, 100 µg/mL. Nuclear DNA was isolated and 8-OH-dG levels were determined by HPLC-ECD. 8-OH-dG levels increased 8- and 14-fold above the control in the10 and 100 g/L NP concentrations, respectively [60]. In another study by Freyria *et al.* human lung epithelial cells (A549) and murine alveolar macrophage (MH-S) cell cultures were incubated in the presence and absence of IONPs. Hematite, in three sizes (nanoparticles, submicrometer particles and microparticles), was used in the study [118]. No DNA damage was induced by hematite nanoparticles in any of the cell lines. Another study was conducted on human alveolar type II-like epithelial cells (A549) and two different types of IONPs. Those particles were Fe_2_O_3_ nanoparticles and microparticles, and magnetite (nanoparticles and microparticles) [28]. In the study, 0.16 million cells were grown in 24 well plates for 24 h, then exposed to 40 and 80 µg/mL of particles for 4 h. Micrometer sized particles were shown to cause more significant DNA damage than the nanometer sized particles. However, cells exposed to 40 µg/mL Fe_3_O_4_ nanoparticle concentrations did show significant oxidative DNA damage.

Karlsson *et al.* exposed type II human lung epithelial cells (A549) to the following NPs: CuZnFe_2_O_4_, Fe_3_O_4_ and Fe_2_O_3_. After exposure, these cells were subjected to the comet assay and no DNA damage was observed for Fe_3_O_4_ or Fe_2_O_3_. However, DNA damage was observed for Fe_3_O_4_ and CuZnFe_2_O_4_ nanoparticles in the form of increased oxidative DNA lesions.

### 2.6. Liver, Kidney and Cerebral Cells

Several investigations were performed to determine the effect of IONPs on mammalian liver cells. Human hepatocytes (HL-7702 cell line) were exposed to Fe_3_O_4_ (magnetite) NPs for 24 h and analyzed with the comet assay [121]. The cells showed nuclear condensation and chromosomal DNA fragmentation after exposure to the NPs. A study by Sadeghi *et al.* investigated human hepatoma Hep G2 cells, which were exposed to Fe_2_O_3_ (75, 100 µg/mL) for 12 and 24 h. Concentration and time dependent DNA damage was observed in Hep G2 cells [53]. In another study conducted by Ma *et al.*, mice were exposed to 2, 10, 20, and 40 mg/kg Fe_3_O_4_ (magnetite) NPs doses for one week. Their hepatic and renal tissues were extracted and analyzed [122]. In liver tissue a significant increase in 8-OH-dG (for the highest dose of NPs) was observed. In kidney tissues, damage was observed for 20 mg/kg NPs dosage. A significant DNA-protein crosslinks (DPC) coefficient in liver tissue at a dose of 40 mg/kg NPs and in kidney tissue at a dose of 40 mg/kg NPs was also observed. A study that used ultra-small superparamagnetic iron oxide nanoparticles (USPIONs, Fe_3_O_4_) and oleic acid coated USPIONs on human cerebral endothelial cells (HCECS) showed single and double DNA strand breaks and alkaline labile sites [123]. A study by Sun *et al.* exposed the following mouse cell lines to aminosilane-coated IONPs (AmS-IONPs) and COOH-AmS-IONPs: brain microvessel endothelial cells, astrocytes, and neurons [124]. None of the particles demonstrated toxicity against mouse brain endothelial cells. However, at high concentrations neurons were damaged by AmS-IONPs and astrocytes were damaged by COOH-AmS-IONPs.

### 2.7. Cancerous Cells

In addition to the mutagenic effects of IONPs, studies have also focused on understanding the genotoxic effects induced by these particles. Apart from healthy cells, several studies have investigated cancerous cells. Different cancer cell types including human fibrosarcoma cells [26], breast cancer cells [52], lung cancer cells [125,126], and cervix cancer cells [127,128] have incorporate IONPs and studied their effects on DNA.

In previous sections IONPs were shown to mediate DNA damage within healthy cells; this is considered problematic. Interestingly, IONPs have also been shown to generate DNA damage within cancerous cells. This observed DNA damage may lead to new cancer treatment methods. As an example Yang *et al.* explore levels of DNA damage in Human fibrosarcoma cells exposed to concentrations of 100, 200, 1000 µg/mL of Fe_3_O_4_ NPs, tetraethyl orthosillicate (TEOS) coated IONP, 3-aminopropyl trimethoxysilane (APTMS) coated IONP and TEOS/APTMS coated IONP. Bare and TEOS coated NPs with cells showed no extensive or dose dependent DNA damage. Another study which used human breast cancer cell line (MCF-7) exposed with Fe_2_O_3_ in different concentrations (0, 10, 30, 60, 120 µg/mL) for 24 and 48 h showed gradual nonlinear DNA damage in a dose and time dependent manner [52]. Two studies showed results for two different lung cancer cell lines: human HCC827 lung cancer cell line and human lung adenocarcinoma type II alveolar epithelial cells A549. Human HCC827 lung cancer cell line was tested with epidermal growth factor receptor (EGFR) targeted hybrid plasmonic core/shell iron oxide gold nanoparticles (225 NP) [126]. In this study western blotting showed a greater increase in phosphorylated histone (γH2AX) indicating DNA strand breaks in 225 nanoparticle treated cells compared with all other cell types. A slight γH2AX increase was observed in AuFe, 225-Ab alone, a mixture of NP and 225-Ab treated cells compared with γH2AX in untreated cells [125,126]. Human cervix carcinoma cells (HeLa cells) and human lung carcinoma cells (A549) were tested with bare Fe_3_O_4_ (magnetite)/SiO_2_ NPs, Fe_3_O_4_ amine-silane surface modified particles and Fe_3_O_4_ sulfonate-silane surface modified particles [127]. HeLa cells and A549 cells were exposed to 5 nM bare and passivated Fe_3_O_4_/SiO_2_ NPs for 48 h. Bare NPs showed increased DNA damage in terms of tail length and DNA percentage in tail relative to passivated NPs (which showed results similar to the control). Another study used HeLa cells (5 × 10^5^ cells/mL), which were cultured in six-well plates. Cells were either unexposed or exposed to NPs (50–200 µg/mL). After exposure to 50 µg/mL NPs, no significant tail size increase was observed. The 100–200 µg/mL concentrations showed an increased tail size and tail DNA% [128].

### 2.8. Bacterial Cells

Interestingly, there is little information in the literature describing the impact of IONPs on bacterial genomes. One such study has been performed using Vero cell line (C1008), bacterial strains *Salmonella typhimurium* (TA98), *S. typhimurium* TA 100, TA 1535, TA 1537, and *E. coli* WP2uvrA 32 with iron (II) and iron (III) oxide (magnetite) NPs [129]. No change in the number of revertant colonies was observed in IONP treated groups and the negative control. Positive controls showed mutagenicity. According to the results IONPs were not mutagenic with respect to bacterial strains *S. typhimurium* TA 100, TA 1535, TA 1537, and *E. coli* WP2uvrA.

An assessment of environmental mutagenicity can only be made in the presence of environmentally significant compounds, such as dissolved organic carbon. Because bacteria are an important component of the environment, the authors sought to expose environmentally significant bacterial cultures to magnetite nanoparticles in the presence and absence of humic acid, after which measures of optical density and DNA damage were collected. To the best of our knowledge, this has not been reported in the literature.

**Figure 6:**
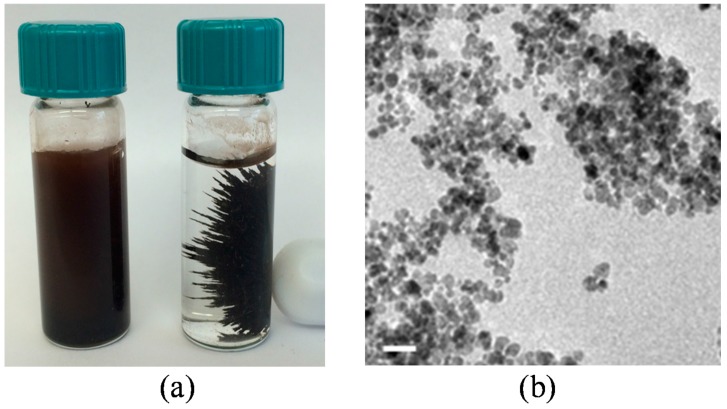
(**a**) Photograph of magnetite IONPs; left vial contains suspended IONPs while the Right vial contains IONPs subjected to a permanent magnet; and (**b**) TEM image of magnetite IONPs with 50 nm scale bar.

Magnetite IONPS with a 10–20 nm diameter were synthesized using the protocol outlined by Petcharoen and Sirivat [130]. A TEM image of our IONPs is shown in Figure 6. Separate and active cultures of *E. coli* and *M. luteus*, grown in TSB, were used in the preparation of the following conditions: (1) control condition: bacteria in TSB; (2) bacteria with IONPs; (3) bacteria with humic acid; and (4) bacteria with both IONPs and humic acid. All bacteria treated with IONPs were exposed to an effective concentration of 4 µg/L. Similarly, all bacteria treated with humic acid were exposed to an effective concentration of 10 mg/L.

**Figure 7:**
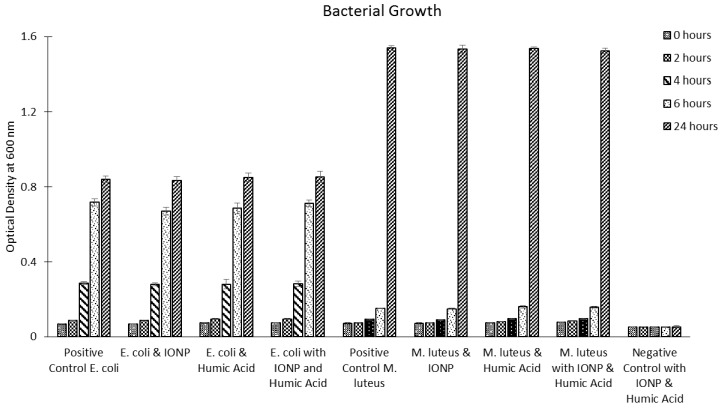
Optical density results for bacterial conditions (*E. coli* and *M. luteus*) over a 24 h period.

**Figure 8:**
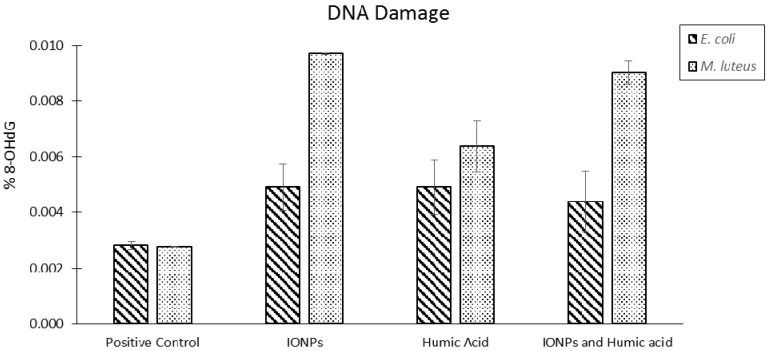
Percentage of DNA damage (as assessed by 8-OHdG levels) for each condition at the 24 h time point.

At time points 0, 2, 4, 6, and 24 h each condition was removed from a rotating incubator (held at 37 °C); five 100 µL samples were taken from each condition and OD_600_ was measured to assess overall bacterial growth. The OD_600_ results are presented in Figure 7. Additionally, DNA damage was measured at the 24 h time point; these results are presented in Figure 8.

A DNA damage kit was used to assess the levels of damage present in each condition at the 24 h time point via the detection of an oxidized derivative of deoxyguanosine, known as 8-hydroxy-2ʹ-deoxyguanosine (8-OHdG). 8-OHdG is considered a marker of oxidative DNA damage, and its presence is known to increase the likelihood of G-to-T transversion mutations during the DNA replication process [131].

Our results demonstrate that IONPs (magnetite) do not impact the growth of *E. coli* or *M. luteus*. All bacterial conditions produced solutions with optical densities equivalent to their respective controls. Based on the optical density data, IONPs do not impact bacterial growth, irrespective of humic acid’s presence. However, the results do indicate that IONPs induce oxidative DNA damage in *M. luteus* (the gram positive model); this oxidative damage appears to be lessened in the presence of humic acid, indicating a protective effect previously shown by source Nick *et al.* [132]. In the case of *E. coli* (the gram negative model), IONPs did not impact the levels of DNA damage, irrespective of humic acid’s presence. From a mechanistic perspective, the authors suspect that the peptidoglycan layer surrounding *M. luteus* mediates an interaction with IONPs triggering oxidative damage of the genomic DNA. Interestingly, in the presence of humic acid this effect is suppressed, possibly because the humic acid is coating the IONPs and altering their interactions with the cellular membrane, effectively conferring a protective layer.

## 3. Summary and Future Perspective

The prevalence of iron in nature, in its various oxidized forms, in combination with low extraction costs, has made finding potential applications for iron oxide nanoparticles (IONPs) highly attractive. Increasingly, novel applications for iron oxides are under investigation as researchers study IONPs as potential drug delivery systems [4], hyperthermia agents [5], magnetic resonance imaging contrast agents [5,6] catalysts for environmental remediation, and much more [7,8,9,10,11,12]. The array of applications associated with IONPs is a result of their tunable properties. There are multiple crystallographic structures exhibited by IONPs [16,17,18,19,20,21,22].

IONPs are widely considered to be non-toxic [39]. However, recent research calls into question the benign nature of IONPs [52,53,54,55]. As increasing numbers of consumer products and industrial processes contain nanoparticles, the unintentional release of these substances into the environment is expected, and the impact of these materials becomes increasingly significant [55,56,57]. The unique properties of nanoscale materials is cause for concern, as their behavior within an environmental setting is unclear. Thus, an assessment of IONP toxicity cannot be made based solely upon the toxicological profile of its bulk counterpart [58]; similarly an assessment of environmental toxicity cannot be made in the absence of environmentally significant compounds, such as dissolved organic carbon. This review article has sought to highlight and add to the literature investigating the impact that IONP exposure has on the genetic components of various cell lines and bacterial strains.

Recent studies have shown that magnetite nanoparticles have the potential to seriously damage healthy cells by inducing oxidative stress or disrupting the existing cytoskeleton [52]. Excess iron has been shown to cause cancer by inducing an overproduction of reactive oxygen species (ROS) capable of damaging cellular components [87]. The Fenton reaction describes one mechanism by which elevated levels of ROS are produced within the cytosol [87]. ROS include: superoxide anion radicals (O^2−^), hydroxyl radicals (**^.^**OH), singlet oxygen (**^.^**O^2^) and hydrogen peroxide (H_2_O_2_) [87]. When DNA is exposed to ROS, 8-hydroxy-2ʹ-deoxyguanosine (8-OHdG) is produced [97]. The presence of this modified of guanosine base increases the likelihood of a G-to-T mutation during DNA replication [131]. Therefore, 8-OHdG acts as a biomarker of oxidative stress, a marker of DNA damage and a site of increased risk for mutagenicity [131].

To date, researchers have found evidence of genotoxic interactions in the following human cell lines, upon exposure to IONPs: lymphoblastoids, fibroblasts, microvascular endothelial cells, bone marrow cells, lung epithelial cells, alveolar type II like epithelial cells, bronchial fibroblasts, skin epithelial cells, hepatocytes, cerebral endothelial cells, fibrosarcoma cells, breast carcinoma cells, lung carcinoma cells, and cervix carcinoma cells. Other cell lines have also shown genotoxic effect upon exposure to IONPs, these cell lines include: Chinese hamster ovary cells, mouse fibroblast cells, murine fibroblast cells, mytilus galloprovincialis sperm cells, mice lung cells, murine alveolar macrophages, mice hepatic and renal tissue cells, and vero cells.

The authors of this review article have come to three primary conclusions regarding the impact of IONPs on biological cells. First, the coating surrounding the IONP has a significant impact on the NPs cellular interaction. As an example, Chen *et al.* (2012) demonstrate that given a single cell line, coated Fe_3_O_4_ NPs induce less DNA damage than do uncoated Fe_3_O_4_ NPs. Similar work, comparing levels of DNA damage induced by coated *vs.* uncoated particles, demonstrate that IONP coatings are capable of either reducing or increasing levels of DNA damage [26,106,108,109,110,111,112,113,123,127]. The second conclusion reached by the authors is that the specific nature of the metallic core impacts a NP’s ability to induce DNA damage. Karlsson *et al.* compared levels of DNA damage caused by uncoated Fe_2_O_3_ and Fe_3_O_4_ NPs, finding that Fe_3_O_4_ NPs produced significant levels of oxidative DNA damage while Fe_2_O_3_ NPs did not [119]. Similarly, Karlsson *et al.* showed that Fe_2_O_3_ NPs did not induce oxidative DNA damage, while Fe_3_O_4_ and CuZnFe_2_O_4_ NPs did induce oxidative DNA damage [63]. The different cellular responses produced by these NPs indicate how the structure and composition of a NP’s metal core impacts its cellular interactions. The third conclusion reached by the authors is that the response to IONPs is highly specific to the cell line under investigation and generalization of IONPs effects is not yet possible. As an example, Watson *et al.* compared levels of DNA damage produced in human lymphoblastoid cells and Chinese hamster ovary cells upon exposure to Fe_2_O_3_ NPs [108]. The researchers found the DNA tail percentages for human lymphoblastiod cells exposed to IONPs to range from 17%–30%, while the DNA tail percentages for IONP treated Chinese hamster ovary cells ranged from 33%–48%. The difference in the levels of DNA damage is suggestive of the poorly understood cell specific interactions produced by IONP exposure. Researchers are finding that the following cell lines have the most dramatic and negative interaction when exposed to IONPs: lung, liver, kidney and sperm cells. These cell lines appear to be most susceptible to DNA damage upon exposure to IONPs [27,28,53,60,115,116,119,120,121,122,123]. Upon exposure to IONPs, cerebral cells are damaged irrespective of the specific IONP coating [123,124]. DNA damage associated with IONP exposure in vascular and blood cells is more complex and appears to be dependent upon the NP’s coating and metallic core. Uncoated Fe_3_O_4_ particles are not shown to induce toxic effects when exposed to lymphoblastoid, leucocyte and bone marrow type blood cells [105,106,107]. In contrast, dextran coated γ-Fe_3_O_4_ and oleic acid coated Fe_3_O_4_ induced toxicity in lymphoblastoid cells [106,107]. However, uncoated Fe_3_O_4_ and Fe_2_O_3_ are toxic toward erythrocytes and lymphoblastoids respectively [104,108]. Interestingly, in fibroblast and stromal cell lines, the NP coating dictates the level of DNA damage induced by IONP exposure. In many cases, coated IONPs caused less DNA damage than did uncoated IONPs [26,110,112]. However, there was a specific coating (3-aminopropyl trimethoxy silane, APTMS), which was shown to cause increased levels of DNA damage [26,113]. IONPs make a significant DNA damage in cancerous cells, irrespective of the coating [52,125,126,127,128]. Unfortunately, there is not enough research in this area to make generalizations about the impact various types of IONP particles on differing cell lines.

The need to understand the behavior of nanoparticles under various environmental settings is essential toward the advancement of nanoscale science and technology [133]. Bacterial cells often serve as good models [134].

Interestingly, there is little information in the literature describing the impact of IONPs on bacterial genomes. The only other articles found which address the mutagenic potential of IONPs with respect to bacterial genomes took into account few bacterial types, and did not attempt to replicate environmental conditions. We sought to expose environmentally significant bacterial cultures to magnetite nanoparticles in the presence and absence of humic acid to assess levels of DNA damage by comparing 8-OHdG levels. We have found that IONPs did induce DNA damage in the gram-positive bacterial model (*M. luteus*), but not in the gram-negative model (*E. coli*). From a mechanistic perspective, the data suggest that interaction between IONPs and the thick peptidoglycan layer of the gram-positive bacteria is responsible for the increased oxidative damage. Furthermore, the data indicates that the presence of humic acid confers protection, by coating the IONP and limiting its interaction with the cell wall. To clarify this result, further studies will need to be conducted which probe the interaction of IONPs with various bacteria in the presence of humic acid.

In summary, we have shown the mutagenic effect of IONPs on various cell lines. This work is significant toward developing a better understanding of the impact of nanoparticles on various cells, whether that interaction is intentional or not. Furthermore, conducting such studies will lead to a proactive approach toward understanding and developing protocols that will lead to guidelines for handling nanoparticles in various environmental settings.
